# Survey of Well-being of Young Children: Implementation and Impact on Care Quality

**DOI:** 10.1097/pq9.0000000000000843

**Published:** 2025-08-27

**Authors:** Yusuke Matsuura, Felix Richter, Gabrielle Block, Carolyn Rosen, Cynthia Katz

**Affiliations:** * Department of Pediatrics, University of Washington, Seattle, WA; † Department of Pediatrics, Seattle Children’s Hospital, Seattle, WA; ‡ Department of Pediatrics, Children's Hospital of Philadelphia, Philadelphia, PA; § Department of Artificial Intelligence, Icahn School of Medicine at Mount Sinai, New York City, NY; ¶ Department of Pediatrics, Icahn School of Medicine at Mount Sinai, New York City, NY.

## Abstract

**Introduction:**

Despite the American Academy of Pediatrics recommendations to use validated developmental screening tools, national adoption remains low. To address this, we launched a quality improvement project to implement the Survey of Well-being of Young Children (SWYC) in our residency clinic and assess its impact. Our primary aim was to achieve more than 50% screening coverage for early childhood well visits, with equitable implementation across both English-speaking and non-English primary speaking families. We also hypothesized that the implementation of SWYC would equitably increase early intervention (EI) referral rates across language groups.

**Methods:**

We included children aged 8–33 months attending well-child visits. Interventions included staff education, daily huddles, and the SWYC integration into the electronic health record. Social work (SW) referrals, though not exclusive to EI, were used as an imperfect proxy, as a designated SW initiates EI referrals in our clinic. We compared referral rates before and after implementation using chi-square tests.

**Results:**

SWYC usage reached 50% within 4 months of implementation. There was no significant difference in SWYC use between English and non-English primary speakers (*P* = 0.131). SW referral rates increased by 2.9 percentage points after implementation (*P* = 0.009), with no significant difference in SW referral rates between language groups (*P* = 0.922).

**Conclusions:**

This quality improvement project highlighted the rapid adoption of a standardized developmental screening tool, resulting in increased SW referrals, which suggests improved identification of developmental concerns with language equity between English and non-English primary speaking families. Standardized screening is an important step in improving developmental outcomes and supporting care equity.

## INTRODUCTION

The prevalence of developmental disabilities is rising, now affecting about 1 in 5 children aged 3–17. These include attention-deficit/hyperactivity disorder, autism spectrum disorder, intellectual disabilities, and delays across various developmental domains.^1,2^The exact reason for this rise remains unclear; however, early detection and intervention of developmental delays are increasingly important for children’s health and future outcomes.^3–7^ The responsibility for early identification falls on general pediatricians. Still, in the absence of standardized screening tools, detection relies on clinical surveillance, which is primarily influenced by time constraints, provider experience, and clinic workflow.

Developmental surveillance involves informal monitoring through history and observation. In contrast, screening involves the use of standardized tools at specific ages, providing quantitative results to identify children who may need further evaluation and intervention. To facilitate early identification, the American Academy of Pediatrics (AAP) recommends validated developmental screening at the 9-, 18-, and 30-month well-child visits.^8,9^ Although these guidelines have increased screening rates from 23% in 2002 to 63% in 2016,^10,11^ national rates remain suboptimal.^12^

The Cohen Center for Pediatric Comprehensive Care at Mount Sinai Kravis Children’s Hospital is an urban academic general pediatrics practice located in the Upper East Side/East Harlem neighborhood of Manhattan in New York City.^13^ The site functions as the main continuity clinic site for the Pediatric Residency Program at the Icahn School of Medicine at Mount Sinai.^14^ It is essential for residents to gain experience in identifying and managing developmental delays during clinic encounters before independent practice.^15^ In our clinic, however, developmental assessments relied on age-appropriate surveillance questions from various sources and lacked the structure of a formal standardized screening tool. Therefore, we decided to implement a standardized, validated developmental screening tool in our general pediatrics practice to identify potential developmental delays in a timely manner, facilitate referrals to appropriate services, and improve long-term outcomes for our patients.

## OBJECTIVES

We implemented a quality improvement (QI) initiative in April 2022 to address a gap in care in our general pediatrics clinic, with the following aims:

Aim 1: To increase the use of a validated screening tool from a baseline of 0% to 50% by June 2024 for well-child visits at 9, 18, 24, and 30 months of age (corrected for prematurity up to 24 mo).
Aim 2: Determine whether implementing a screening tool increases referral rates to early intervention (EI) services.

Additionally, the initiative aimed to ensure equitable screening practices for both English-speaking and non-English primary speaking patients. We selected the age range for aim 1 to align with AAP guidelines and the eligibility criteria for EI services.

## METHODS

### Setting

We conducted this QI project at the Cohen Center for Pediatric Comprehensive Care, the pediatric resident continuity clinic at the Mount Sinai Hospital in New York City. Our medium-sized residency program includes categorical and combined programs (child neurology, pediatric genetics, and triple board). The clinic’s catchment area spans all 5 New York City boroughs and surrounding regions, including Westchester and New Jersey. With approximately 2,000 well-child and follow-up visits monthly, about 200 involve children in the study’s target age groups (8 and 33 mo). The QI team included 13 residents and 2 supervising attendings. This project was approved and classified as a QI initiative by the institutional review board and was deemed exempt from formal institutional review board review.

### Literature Review and Determination of Implemented Tool

In July 2022, we conducted a comprehensive PubMed literature review of more than 30 studies to identify a validated developmental screening tool. A key study, Comparative accuracy of developmental screening questionnaires, published in Journal of the American Medical Association ,^16^ compared the Ages and Stages Questionnaires, Third Edition (ASQ-3); the Survey of Well-being of Young Children (SWYC); and the Parents’ Evaluation of Developmental Status (PEDS).

The study reported specificities for younger children (0–42 mo) of 89.4% (ASQ-3), 89.0% (SWYC), and 79.6% (PEDS), and sensitivities for severe delays of 60.0%, 73.7%, and 78.9%, respectively. The ASQ-3 and SWYC showed significantly higher specificity than the PEDS (*P* < 0.001 and *P* = 0.002, respectively). However, sensitivity differences were not statistically significant. Sensitivity exceeded 70% only in cases of severe delays, particularly when comparing the SWYC and PEDS in younger children. Based on content, completion time, feasibility, and cost, we selected the SWYC.

The SWYC, developed at Tufts Medical Center, is a comprehensive, freely available developmental screening tool for pediatric primary care settings.^17,18^ It addresses motor, language, and social-emotional development, as well as caregiver concerns about behavior and family stressors. For this QI initiative, we decided to implement the SWYC developmental milestones questionnaire section, which includes 10 caregiver-rated questions assessing language, gross motor, fine motor, and personal-social skills. Caregivers are asked to select “not yet (0),” “somewhat (1),” or “very much (2)” to generate a score indicating whether the child “appears to meet age expectations” or “needs review.” As an example, the SWYC questionnaire for 9-month-olds is available at the following links: the questionnaire itself (https://www.tuftsmedicine.org/sites/default/files/2024-01/9-Month-English-Age-specific-2021.pdf) and the score interpretation sheet (https://www.tuftsmedicine.org/sites/default/files/2024-01/scoring-cheat-sheet-v2.pdf).

**Fig. 1.**
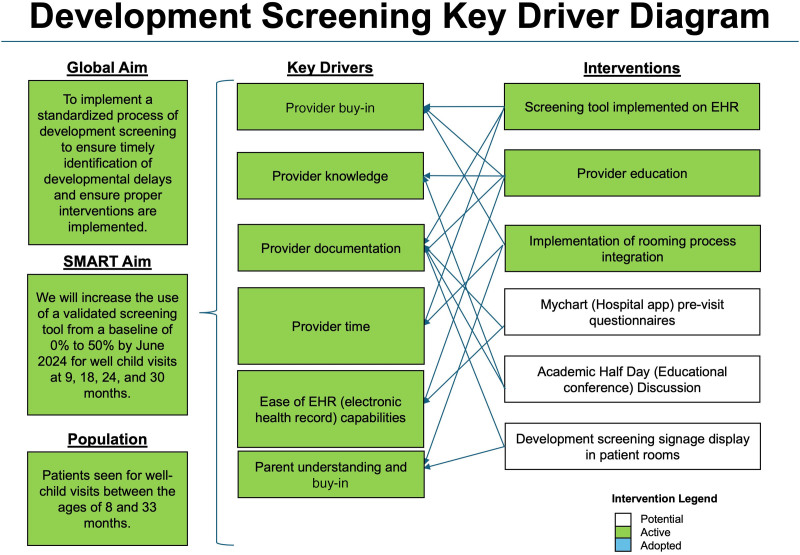
Developmental screening implementation key driver diagram.

Following the selection of the screening tool, we used QI tools to support planning and implementation. The process map visualized each step and clarified how implementation should occur within the practice workflow.^19,20^ We used a fishbone diagram to identify potential barriers to implementing the SWYC screening in the clinic and facilitate the development of a key driver diagram.^21,22^ The key driver diagram helped identify appropriate and feasible interventions to achieve our aim (Fig. 1).^23,24^

### Inclusion/Exclusion Criteria

The study population included all patients aged 8 and 33 months attending well-child visits at the Cohen Center for Pediatric Comprehensive Care (corrected for gestational age through 24 mo). Exclusion criteria included patients seen for urgent visits, follow-up visits, or well-child visits for those younger than 7 months or older than 34 months of age.

### Outcome/Process/Balancing Measures

The initial outcome measure was the percentage of patients aged 8–33 months presenting for well-child visits with a completed SWYC questionnaire.

The process measure was the percentage of patients who completed a paper SWYC questionnaire before the physician entered the room. As the Epic platform at Mount Sinai had not yet integrated the SWYC into the patient portal (MyChart), paper forms in English and Spanish were distributed at check-in during the pilot phase (November 2022). Spanish paper forms were used as the non-English pilot language due to their high prevalence among our patient population. Several challenges arose, including maintaining paper form supplies, incomplete responses, and difficulties identifying eligible visits and assigning age-appropriate forms. These challenges led to the discontinuation of the paper-based system and a shift to providers completing the SWYC within the electronic health record (EHR) during the visit.

The SWYC is originally intended for caregivers to complete before the visit. However, the SWYC user’s manual also permits provider-administered screening under specific conditions, including verbal administration in a private setting and adherence to the form’s wording.^25^ Our clinic followed these recommendations.

As a potential balancing measure in the QI protocol, we considered tracking In-Training Examination scores due to concerns about reduced active learning; however, this was not pursued due to privacy restrictions on accessing In-Training Examination data.

### Pilot Implementation of EHR-integrated SWYC Screening

Before the pilot phase, we pursued the integration of the SWYC into Epic, the EHR used in our healthcare system. With the help of our local Epic information technology (IT) team, the SWYC was successfully embedded within outpatient encounters. Providers could access the SWYC tab in the Rooming tab, where questions and responses were integrated for direct input. Each questionnaire was linked to the patient’s corrected age and automatically generated a score upon completion.

The EHR-integrated pilot launched on November 14, 2022, with several team members testing SWYC during patient encounters. Simultaneously, as a process measure, the team distributed SWYC paper forms for patients to complete before the provider entered the room, which was impractical. As a result, we transitioned to having providers complete the SWYC screening directly within the EHR during well-child visits. Our team informally tracked the time to complete and document questionnaires. Before the project, developmental assessments used informal surveillance questions, which were later replaced by the SWYC. Both required similar levels of provider engagement (typically 1–3 min), and encounter durations seemed comparable, even when using an interpreter, based on observations.

Technical issues were reported to the Epic support team, and adjustments were made accordingly. By February 2023, all major issues had been resolved, and the system was ready for broader dissemination.

### Dissemination of the SWYC

In February 2023, our project team launched the EHR-integrated SWYC tool via email campaigns, accompanied by visual guides, to pediatric residents and outpatient faculty. To document SWYC results in the Epic note, all providers were instructed to open the Exam Room tab within the Rooming tab, scroll to the “Survey of Well-being of Young Children (SWYC)” section, then copy and paste the questions and responses into the developmental section of the note manually. Further interventions included a follow-up email reminder in March 2023 and the addition of SWYC screening reminders to daily clinic huddle announcements beginning in April 2023.

In line with our established workflow, EI referrals were made when patients were identified as at risk of developmental delay by using the referral tab to request an ambulatory social worker (SW). Throughout the project, several educational efforts supported implementation, including a residency-wide presentation and additional instructional emails in June 2023. In July 2023, a new group of pediatric residents at Mount Sinai Hospital received training on the developmental screening workflow during their orientation.

During this phase, we worked closely with the IT team to develop a dot phrase that would simplify the input of SWYC results into the note. This tool was fully implemented in September 2023, accompanied by reminder emails and huddles. Using the dot phrase. “SWYC—months,” providers can automatically pull SWYC questionnaire results and interpretations into the patient’s encounter note. With full EHR integration, the SWYC results, scores, and an assessment of either “appears to meet age expectations” or “needs review” were made available for direct documentation in the note.

### Data Collection

During the dissemination phase, we secured access to an automated report provided by the IT team, which included patient demographics (name, medical record number, birth date, preferred language, and insurance coverage), as well as well-child visit date, age at visit, provider name, SWYC score and assessment, and the date of SW referral. This report was sent to QI team members monthly in an Excel spreadsheet to support the auditing of our outcome measures and to guide improvement strategies.

### Referrals Data Collection and Analysis

We evaluated its secondary impact on patients, specifically focusing on referrals to SW for potential EI. In our clinic, 4–5 SWs are typically present, but 1 designated SW is responsible for initiating the EI referral process. We collected data to assess changes in SW referrals following the implementation of SWYC. However, because the data did not capture the specific reason for each SW referral, we were unable to isolate EI-specific referrals. As such, although the overall SW referrals served as a proxy for the process measure, we acknowledge that they may not accurately reflect the actual rate of EI referrals.

We extracted monthly data from April 2022 until November 2023. We excluded patients for whom an SW referral had already been made before the encounter, as including those patients would not accurately reflect referrals due to our interventions because each visit’s data in the Excel spreadsheet only recorded the first date of an SW referral, which could have led to an overestimation of referral rates if the same patient visited the clinic multiple times. We calculated the monthly SW referral rate by dividing the number of new referrals by the total number of eligible patient encounters.

We used a P-chart, a type of statistical process control chart, to display monthly referral trends. The centerline was initially set based on the average referral rate before the start of implementation. Following standard statistical process control rules, the centerline was adjusted when 8 or more consecutive points fell above or below the previous centerline, indicating a significant process shift. Control limits were calculated using the binomial distribution and adjusted for varying monthly sample sizes.

We defined the pre-SWYC period as April–November 2022 and the post-SWYC period as April–November 2023, when SWYC performance exceeded 50%. Referral rates between the 2 periods were compared using a chi-square test in R software.

### Language Equity Data Collection and Analysis

Finally, to ensure equity, we evaluated whether there was a significant difference in (1) SWYC performance rates and (2) SW referral rates during the post-SWYC period between patients whose primary language was English and those whose preferred language was not English. Non-English primary language was determined based on the preferred language recorded in Epic, which is entered either by patients through MyChart or during onsite registration. Chi-square tests were conducted in R software to compare the 2 language groups.

## RESULTS

### Result 1: SWYC Performance Rate

**Fig. 2.**
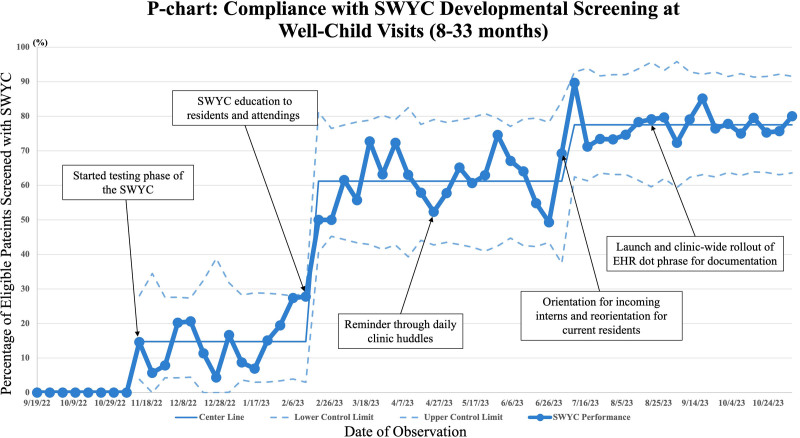
P-chart: the SWYC performance trend, annotated with major events.

Before implementation, the baseline SWYC screening rate was 0%. Compliance with screening was plotted on an annotated P-chart showing incremental success resulting from our interventions (Fig. 2). Compliance first exceeded our goal of 50% 4 months after the implementation of the SWYC.

### Result 2: SW Referral Rate

**Fig. 3.**
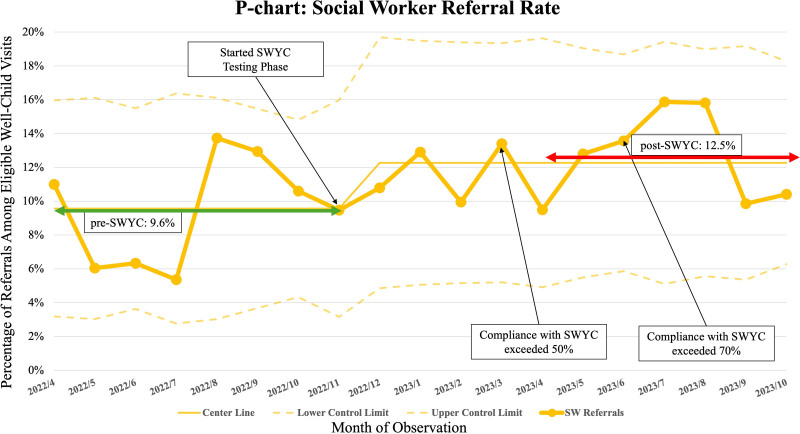
P-chart: SW referral rates trend, annotated with major events.

Our EHR data collection included tracking of SW referrals (as a proxy measure of EI referrals). Figure 3 presents a P-chart of SW referrals over time. The *y* axis represents the percentage of SW referrals among the total eligible visits. The pre-SWYC SW referral rate (April–November 2022) was 9.6%, whereas the post-SWYC referral rate, measured from April to November 2023, during which the SWYC completion rate exceeded 50%, increased to 12.5%. The resulting *P* value of 0.009 (odds ratio [OR]: 1.35, 95% CI: 1.08–1.67) indicates a statistically significant difference (Table 1).

**Table 1.** Number of SW Referrals Pre-SWYC (April–November 2022) and Post-SWYC Period (April–November 2023), and Assessment of Statistical Significance

	Pre-SWYC (April–November 2022)	Post-SWYC (April–November 2023) (SWYC Performance > 50%)	*P*
SW referral +	157	217	0.009
SW referral −	1,484	1,524	

### Result 3: Language Equity in SWYC Performance and SW Referrals

During the post-SWYC period, there was no difference in SWYC performance rates between patients identifying English as their primary language and those identifying non-English as their primary language (*P* = 0.13; OR: 1.27, 95% CI: 0.93–1.72) (Table 2). Similarly, there was no difference in SW referral rates between the 2 language groups following SWYC implementation (*P* = 0.9; OR: 0.96; 95% CI: 0.63–1.45) (Table 2).

**Table 2.** Comparison of SWYC Completion Rates and SW Referral Rates Between English and Non-English Primary Language Speakers During the Post-SWYC Period (April–November 2023) and Assessment of Statistical Significance

Post-SWYC (SWYC Performance > 50%)	SWYC Done	SWYC Not Done	*P* (SWYC)	SW Referral+	SW Referral −	*P* (SW)
English primary speaker	1,219	405	0.131	188	1,328	0.922
Non-English primary speaker	173	73		29	196	

## DISCUSSION

Given the well-documented benefits of EI in improving therapy outcomes and long-term developmental progress,^3–7^ early identification of developmental delays during well-child visits and connecting children to EI services^26^ is crucial. However, integrating a new developmental screening system within a healthcare framework is inherently complex.

An AAP survey of 688 pediatricians found that 41% did not use standardized screening tools due to time constraints (46%) and lack of EHR integration (39.9%), which led to reliance on paper forms and manual entry. Additionally, 26.2% of parents declined to complete the forms, which limited screening uptake.^27^ Similar challenges have been reported in clinics including persistently low documentation rates (40%) even after the implementation of the standardized screening, PEDS, primarily due to paper-based forms and manual EHR entry,^28^ and a 40% nonreturn rate of mailed ASQ and Modified Checklist for Autism in Toddlers questionnaires,^29^ reflecting issues with caregiver engagement. These challenges underscore the difficulties of implementing systematic change within clinical practice.

Despite these common obstacles, our QI project achieved screening rates greater than 50% within 4 months of implementation with equitable screening practices for both English-speaking patients and those for whom English is not the primary language. We believe the success of our QI project can be attributed to several key factors.

### EHR Integration

A key improvement was the automated integration of the SWYC into the EHR system, eliminating the need for paper forms. During the pilot phase, several barriers to paper-based screening were identified while evaluating process measures, including

- unclear distribution protocols, including a lack of clear guidance on which handouts to provide based on the child’s age and language, creating confusion for front desk staff;
- logistical issues, such as ensuring pens were available and parents had dedicated spaces and time to complete while waiting;
- manual data entry burden, requiring providers to transcribe responses into the EHR, adding to their workload.

By identifying these challenges early in the process, we transitioned to a fully digitized screening system within the EHR, allowing providers to complete the SWYC seamlessly during encounters, thereby streamlining the workflow and reducing administrative burden.

### Practical Scoring and Recommendations in the SWYC

The SWYC is designed to be time-efficient and user-friendly. Its 10-question format and straightforward scoring system made it practical for providers, whereas its built-in management recommendations further supported decision-making. These features not only reduced provider burden but also enhanced confidence in identifying and referring patients to EI services.

### Plan–Do–Check–Act Cycle

To support ongoing improvement, we used iterative Plan–Do–Check–Act cycles and implemented multiple interventions. Provider engagement strategies, including emails, presentations, and daily huddles, played a critical role in the rapid adoption of SWYC. These interventions kept providers informed and engaged, contributing to a swift increase in screening compliance and sustaining improvements over time.

This study has several limitations. First, we cannot confirm that all the SW referrals were exclusively for EI referrals, as some may have been made for other needs, such as housing and food insecurity. This limitation arises from the inability to distinguish between referral reasons within the available data. However, based on a general review of referral patterns in our clinic, informed by provider input, most referrals for children aged 6 months to 3 years were for EI evaluations. Additionally, our clinic’s workflow includes a designated SW responsible for initiating EI referrals, which supports the use of overall SW referral rates as a reasonable, though imperfect, proxy process measure. Second, there are limitations related to the generalizability of EHR use. Although many practices are transitioning to electronic records, not all outpatient settings have adopted EHRs. Moreover, integrating the SWYC into the EHR may not be straightforward or feasible in all record systems.^27^

To sustain the project, it is crucial to continue emphasizing the importance of consistent SWYC screening to providers and to monitor screening performance rates to improve compliance rates further.

## CONCLUSIONS

A new validated developmental screening tool using the SWYC was successfully implemented in a residency continuity clinic through EHR integration, educational sessions, and regular reminders. Its use likely enabled the timely identification of developmental delays. It facilitated appropriate referrals to SW for potential EI evaluations, supported by the SWYC’s built-in scoring and recommendation system. Implementing a standardized developmental screening system ideally integrated into the EHR is an important step in improving developmental outcomes for children in the United States and supporting care equity.
